# Supplementary material for: Associations between serum uric acid and depression in U.S. adults: An analysis of the national health and nutrition examination survey 2007–2016
Source: PLoS One. 2025 Apr 8;20(4):e0321387. doi: 10.1371/journal.pone.0321387 (PMC11978101; doi:10.1371/journal.pone.0321387)
Supplement: Attachment — Submitted filename: PONE-D-24-31772_reviewer.pdf [file pone.0321387.s001.pdf]

PLOS ONE

Associations between serum uric acid and depression in U.S. adults: an analysis of the National Health and Nutrition Examination Survey 2007–2016

--Manuscript Draft--

**Manuscript Number:**	PONE-D-24-31772
**Article Type:**	Research Article
**Full Title:**	Associations between serum uric acid and depression in U.S. adults: an analysis of the National Health and Nutrition Examination Survey 2007–2016
**Short Title:**	Associations between serum uric acid and depression in U.S. adults
**Corresponding Author:**	zhang ertao<br>Changzhi Medical College<br>中国, 山西省大同市 CHINA
**Keywords:**	serum uric acid; depression; gender; oxidative stress; diabetes
**Abstract:**	Limited research has specifically explored the gender-specific in relation to serum uric acid and depression. Therefore, the research aims to bridge that gap by exploring the relationships between serum uric acid and depression, stratified by gender, in U.S. adults. In this study, the participants comprised of individuals from five consecutive survey cycles (NHANES 2007-2008 to 2015-2016), 20804 qualified participants were incorporated into the research. The assessment of depression was conducted utilizing the Patient Health Questionnaire-9 (PHQ-9), We calculated the weighted averages (95% CIs) for continuous variables and the percentages (95% CIs) for categorical variables among the chosen covariates. A multivariable logistic regression model with weights was utilized to assess the relationships between serum uric acid categories and depression status, In male subjects, uric acid levels may be linked inversely to depression. However, Tertile3(≥303.3 μmol/L) was more likely to develop depression than Tertile1 (<249.8 μmol/L) among women .The study show that high serum uric acid level may reduce the incidence of depression in men, but in female, it as the independent factors that increase the risk of depression.
**Order of Authors:**	zhang ertao
**Additional Information:**	
**Question**	**Response**
**Financial Disclosure**<br><br>Enter a financial disclosure statement that describes the sources of funding for the work included in this submission. Review the submission guidelines for detailed requirements. View published research articles from *PLOS ONE* for specific examples.<br><br>This statement is required for submission and **will appear in the published article** if the submission is accepted. Please make sure it is accurate.	The author(s) received no specific funding for this work.

**Funded studies**<br>Enter a statement with the following details:<br>• Initials of the authors who received each award<br>• Grant numbers awarded to each author<br>• The full name of each funder<br>• URL of each funder website<br>• Did the sponsors or funders play any role in the study design, data collection and analysis, decision to publish, or preparation of the manuscript?<br><br>Did you receive funding for this work?	
**Competing Interests**<br><br>Use the instructions below to enter a competing interest statement for this submission. On behalf of all authors, disclose any competing interests that could be perceived to bias this work—acknowledging all financial support and any other relevant financial or non-financial competing interests.<br><br>This statement is required for submission and **will appear in the published article** if the submission is accepted. Please make sure it is accurate and that any funding sources listed in your Funding Information later in the submission form are also declared in your Financial Disclosure statement.<br><br>View published research articles from *PLOS ONE* for specific examples.	The authors have declared that no competing interests exist.

**NO authors have competing interests**<br><br>Enter: *The authors have declared that no competing interests exist.*<br><br>**Authors with competing interests**<br><br>Enter competing interest details beginning with this statement:<br><br>*I have read the journal's policy and the authors of this manuscript have the following competing interests: [insert competing interests here]*<br><br>* typeset	
**Ethics Statement**<br><br>Enter an ethics statement for this submission. This statement is required if the study involved:<br><br>• Human participants<br>• Human specimens or tissue<br>• Vertebrate animals or cephalopods<br>• Vertebrate embryos or tissues<br>• Field research<br><br>Write "N/A" if the submission does not require an ethics statement.<br><br>General guidance is provided below. Consult the submission guidelines for detailed instructions. **Make sure that all information entered here is included in the Methods section of the manuscript.**	N/A

Powered by Editorial Manager® and ProduXion Manager® from Aries Systems Corporation

**Format for specific study types**<br><br>**Human Subject Research (involving human participants and/or tissue)**<br>• Give the name of the institutional review board or ethics committee that approved the study<br>• Include the approval number and/or a statement indicating approval of this research<br>• Indicate the form of consent obtained (written/oral) or the reason that consent was not obtained (e.g. the data were analyzed anonymously)<br><br>**Animal Research (involving vertebrate animals, embryos or tissues)**<br>• Provide the name of the Institutional Animal Care and Use Committee (IACUC) or other relevant ethics board that reviewed the study protocol, and indicate whether they approved this research or granted a formal waiver of ethical approval<br>• Include an approval number if one was obtained<br>• If the study involved *non-human primates*, add *additional details* about animal welfare and steps taken to ameliorate suffering<br>• If anesthesia, euthanasia, or any kind of animal sacrifice is part of the study, include briefly which substances and/or methods were applied<br><br>**Field Research**<br><br>Include the following details if this study involves the collection of plant, animal, or other materials from a natural setting:<br>• Field permit number<br>• Name of the institution or relevant body that granted permission	
**Data Availability**<br><br>Authors are required to make all data underlying the findings described fully available, without restriction, and from the time of publication. PLOS allows rare exceptions to address legal and ethical concerns. See the PLOS Data Policy and FAQ for detailed information.	Yes - all data are fully available without restriction

A Data Availability Statement describing where the data can be found is required at submission. Your answers to this question constitute the Data Availability Statement and **will be published in the article**, if accepted.<br><br>**Important:** Stating 'data available on request from the author' is not sufficient. If your data are only available upon request, select 'No' for the first question and explain your exceptional situation in the text box.<br><br>Do the authors confirm that all data underlying the findings described in their manuscript are fully available without restriction?	
**Describe where the data may be found in full sentences. If you are copying our sample text, replace any instances of XXX with the appropriate details.**<br><br>• If the data are **held or will be held in a public repository**, include URLs, accession numbers or DOIs. If this information will only be available after acceptance, indicate this by ticking the box below. For example: *All XXX files are available from the XXX database (accession number(s) XXX, XXX.*).<br>• If the data are all contained **within the manuscript and/or Supporting Information files**, enter the following: *All relevant data are within the manuscript and its Supporting Information files.*<br>• If neither of these applies but you are able to provide **details of access elsewhere**, with or without limitations, please do so. For example:<br><br>*Data cannot be shared publicly because of [XXX]. Data are available from the XXX Institutional Data Access / Ethics Committee (contact via XXX) for researchers who meet the criteria for access to confidential data.*<br><br>*The data underlying the results presented in the study are available from (include the name of the third party*	The NHANES database is publicly available (http://www.cdc.gov/nchs/nhanes.htm).

and contact information or URL).<br>• This text is appropriate if the data are owned by a third party and authors do not have permission to share the data.<br><br>* typeset	
Additional data availability information:	Tick here if the URLs/accession numbers/DOIs will be available only after acceptance of the manuscript for publication so that we can ensure their inclusion before publication.

Manuscript

Click here to access/download;Manuscript;uric acid and depression.docx.docx

Associations between serum uric acid and depression in U.S. adults: an analysis of the National Health and Nutrition Examination Survey 2007–2016

Abstract

Limited research has specifically explored the gender-specific in relation to serum uric acid and depression. Therefore, the research aims to bridge that gap by exploring the relationships between serum uric acid and depression, stratified by gender, in U.S. adults. In this study, the participants comprised of individuals from five consecutive survey cycles (NHANES 2007-2008 to 2015-2016), 20804 qualified participants were incorporated into the research. The assessment of depression was conducted utilizing the Patient Health Questionnaire-9 (PHQ-9), We calculated the weighted averages (95% CIs) for continuous variables and the percentages (95% CIs) for categorical variables among the chosen covariates. A multivariable logistic regression model with weights was utilized to assess the relationships between serum uric acid categories and depression status, In male subjects, uric acid levels may be linked inversely to depression. However, Tertile3(≥303.3 μmol/L） was more likely to develop depression than Tertile1（<249.8 μmol/L） among women .The study show that high serum uric acid level may reduce the incidence of depression in men, but in female, it as the independent factors that increase the risk of depression.

KEYWORDS

serum uric acid; depression; gender; oxidative stress; diabetes

Aims

The aim of the study is to explore the correlation between serum uric acid levels and depression, taking into account gender differences in U.S adults.

The findings from past research on the associations between serum uric acid levels, depression, and gender are limited. And previous studies have demonstrated a link to serum uric acid levels and depression. However, only little number of these studies have specifically found the gender-specific associations between serum uric acid and depression. Consequently, our objective was to delve into the relationships with serum uric acid and depression, considering gender differences in the United States adults

Introduction

Serum uric acid is the end product resulting from the breakdown of purine nucleotides, specifically adenine and guanine, it's primarily known for its association with hyperuricemia.(1)In the recent years, the sickness rate of hyperuricemia has increased yearly with the development of life. And in 2020, the number of people with hyperuricemia in the world was about 930 million. serum uric acid is an important antioxidant, contributing over more than 60% of the total antioxidant capacity in plasma,(2) therefore it could be especially significant in depression.

Antioxidants are essential in neutralizing reactive oxygen species (ROS) and free radicals, safeguarding cells from oxidative stress (a process caused by damage to cellular structures due to decreased antioxidant activity). The brain, consuming 20% of the body's oxygen, produces significant amounts of ROS, making it particularly susceptible to oxidative stress.(3)When it comes to serum uric acid, it exhibits dual behavior. While it can act as a pro-oxidant inside cells, a significant number of studies have revealed a positive correlation between serum uric acid and chronic diseases.(4-6) On the contrary, in the extracellular chambers, the main function of serum uric acid is

antioxidants. In fact, previous research has indicated that serum uric acid contributes to approximately 60% of the antioxidant capacity in body's blood.[7]Furthermore, studies have linked elevated levels of serum uric acid are associated with reduced rates of depression and anxiety.[8, 9]It appears that gender plays a role in the relationship between serum uric acid and depression. Although evidence suggests serum uric acid was associated with depression in a negative manner, few studies have explored this relationship specifically in relation to gender. Prior studies have shown that women are more prone to depression than men, but men tend to exhibit higher levels of uric acid compared to female.[10] However, current research indicates that serum uric acid has a negative effect on male depression, but not on women.[11]

The level of uric acid in participants with depression is lacking a unified consensus. the study reported the control group was found to have lower levels of serum uric acid compared to patients with depression.[9]However, other studies suggest that due to increased antioxidative stress, serum uric acid levels may decrease in depression.[12]This discrepancy can be attributed to several factors. Firstly, depression exhibits various clinical subtypes, and the serum uric acid levels may vary among these subtypes, which has not been considered in previous studies. Secondly, published studies on depression utilize diverse diagnostic criteria. The Patient Health Questionnaire (PHQ-9) is widely regarded as the standard diagnostic tool for depression. It's crucial to note that significant differences exist in the diagnostic criteria of depression across different classifications. The current study focuses on patients diagnosed with depression using PHQ-9 criteria upon discharge, and aiming to explore uric acid levels, depression, and sexual activity can be interconnected in the

United States.

Methods and materials

Study design and subjects

The National Institute of Health Statistics (NCHS), which is managed by the National Center for Health Statistics (NCHS) of the Centers for Disease Control, is a continuous, multi-departmental national survey of the civilian population of the United States. It adopts a complex, multistage sampling design to include a representative sample of about 10 thousand individuals in each survey cycle. A detailed description of the NHANES database is publicly available (http://www.cdc.gov/nchs/nhanes.htm). Written and informed consent is the basis for the Institutional Review Committee of the National Centre for Health Statistics approving the NHANES program.

The present research included the participants from five consecutive survey cycles (NHANES 2007-2008 to 2015-2016). Firstly, participants were excluded if they were pregnant or uncertain about their pregnancy status during the check-up (n=715), or were under 18 years old (n=19,864). The subjects of missing data in serum uric acid and depression status were excluded (n = 4401). In the end, the research encompassed a total of 20,804 qualified participants.

Data collection

The assessment of depression was conducted utilizing the Patient Health Questionnaire-9 (PHQ-9),a nine-item screening instrument that asks questions about the frequency of symptoms of depression over the past 2 weeks. Response categories "not at all," "several days," "more than half the days" and "nearly every day" were

given a score ranging from 0 to 3. A total score was calculated ranging from 0 to 27. A score of 10 or higher has been well validated and is used to define depression.

To determine the concentration of serum uric acid in serum, plasma, or urine, a timed endpoint method was employed. This method involves the oxidation of uric acid by uricase, resulting in the formation of allatoin and hydrogen peroxide. Subsequently, the hydrogen peroxide reacts with 4-aminoantipyrine (4-AAP) and 3,5-dichloro-2-hydroxybenzene sulfonate (DCHBS) in the presence of peroxidase, yielding a colored product. The system measures the change in absorbance at a specific wavelength of 520 nm at regular intervals. It's worth noting that the change in absorbance directly correlates with the concentration of serum uric acid present in the sample.

The survey was conducted by well-trained interviewers through questionnaires to collect information on demographic characteristics, life style factors and present prescription drugs. The demographic characteristics included sex (men, women), age, ethnicity (Mexican American, other Hispanic, non-Hispanic white and non-Hispanic black), education (lower than high school, high school or above), annual household income (low income < \$45,000, high income ≥ \$45,000). The Global Physical Activity Questionnaire was utilized to estimate physical activity by inquiring about the subjects' physical activity intensity, duration, and frequency. The level of physical activity was then measured by calculating the total metabolic equivalents minutes per week. The physical examinations of NHANES is carried out in a mobile physical examination center, using standardized protocols and calibration

equipment, The detailed information of data collection is available on the website(https://wwwn.cdc.gov/nchs/nhanes/analyticguidelines.aspx).

Statistical analysis

During the analysis, we took into consideration the varying probabilities of selection, the intricate sample design, as well as nonresponse and noncoverage by employing sample weights. We computed the weighted means along with their respective 95% CIs for continuous variables, and percentages along with their 95% CIs for categorical variables of the chosen covariates. Standard errors were determined through the linearization of Taylor series. To delineate the non-linear relationships between serum uric acid and depression, we employed restricted cubic splines with three knots placed at the 5th, 50th, and 95th percentiles. A multivariable logistic regression model with weights was utilized to assess the relationships between serum uric acid categories and depression status, taking into account appropriate adjustments for age, education level, physical activity level, smoking ,drinking status, income, creatinine, race/ethnicity, body mass index, and albumin-to-creatinine ratio. A statistical significance level of $P < 0.05$ was deemed appropriate. All the statistical analysis was conducted using survey package in R version 4.0.3 (http://www.r-project.org).

Results

Table 1 lists the general characteristics of the research population. The analysis encompassed a total of 20804 participants. (9096 men and 9500 women),6.25% (568) male and 4.32% (410) female endorsing depression symptoms (i.e., PHQ-9≥5). Male and female with an average age of 42 years. And it's obvious that participants with

depression symptoms tend to be smokers, drinkers, Additionally, they are more likely to have less education, lower income and less met-activity from Table 1. For both sexes, participants have higher acr and GGT, it shows that people with kidney disease increased vulnerability to depression. And there are 7.98%（725）men are prediagDM2, 13.97%（1327）women are prediagDM2.)

As shown in Figure 1, the serum uric acid level has a linear relationship with depression and gender by Generalized Additive Models. Whether serum uric acid goes up or down, it increases the risk of depression in women. However, for man, there is a protective effect against depression when serum uric acid increased approximately to more than 320umol/L. In Table 2, in males, serum uric acid levels in Tertile1(<327.1μmol/L), are not associated with depression, and as serum uric acid levels rise, the incidence of depression is lower and protective against depression. However, in female group with diabetes, when serum uric acid is at Tertile2(249.8~303.3μmol/L), indicated that serum uric acid has nothing to do with depression, but when serum uric acid is greater than or less than this level, the risk of depression is higher. When compared with the 1st quartile of serum uric acid index, patients with multivariate adjusted OR in the 3rd quartile of serum uric acid index are tend to suffer from depression (OR = 1.97; 95% confidence interval (CI) 1.24, 3.13). Both men and women with diabetes are more prone to depression. Age, education level, degree of physical activity, smoking habits, alcohol consumption, and income level, creatinine, race/ethnicity, body mass index, and albumin-to-creatinine ratio may be is an independent risk factor for depression.

Table 3 showed that in male subjects, serum uric acid levels in Tertile 2 (327.1~386.6μmol/L) and Tertile 3 (≥386.6μmol/L) did not increase the risk of depression, There may be no connection between serum uric acid and male depression. However, among female, Tertile 3(≥303.3μmol/L） was more likely to develop depression than Tertile 1 （<249.8μmol/L） , as can be seen in table 4. Both tables show that people with diabetes have a higher risk of depression.

Discussion

This national representative cross-sectional study has showed a link between serum uric acid level and depression and gender. Paucity of studies examining the gender relationship between uric acid and depression. Thus, we stratified they relationship by gender to explore it, and found a meaningful result. According to this study, we search that higher serum uric acid has a correlation with a higher risk of depression in American female population, but it's opposite in the male.

Earlier study showed that men tend to exhibit higher levels serum uric acid in comparison to women, yet surprisingly, they exhibit a lower prevalence of depression, it's similar to our found.(13)However, Yaru Li's study indicated a negative correlation between serum uric acid levels and the prevalence of depression among men, but has nothing to do with the prevalence of depression in women,(10)and Meng's analysis showed that depressed patients have lower serum uric acid compare to normal population whether man or woman.(12) In addition, a Korea survey found that the prevalence of depressive symptoms was elevated in women with lower serum uric acid levels than in those with higher serum uric acid levels and it's no significant association between serum uric acid levels and depressive symptoms was observed in

men. [14]the reasons for this may be that we use the different depression scales, there is significant variation in the diagnostic criteria for depression among different classifications. Different race and life style may cause the different results too.

The mechanism of serum uric acid and depression is not clear, uric acid is an antioxidant enzyme, and has different physiological effects in the occurrence and development of different diseases. Under normal physiological conditions, serum uric acid can interact with a variety of oxidants, including hydrogen peroxide and hydroxyl free radicals, to clear oxygen free radicals, inhibit lipid peroxidation, avoid oxidative damage, and effectively play a neuroprotective role.[15, 16]But other study shows that low serum uric acid level in central nervous system may damage the antioxidant capacity of cells.[17] Levels of uric acid and purines, like adenosine, play a role in regulating mood, sleep, activity, appetite, cognition, memory, susceptibility to seizures, social interaction, and impulsivity.[18] The inhibition of A1 receptor and the activation of A2A receptor are influenced by purine, and purinergic signal plays a pivotal role in the pathophysiology of depression.[19] serum uric acid is the final product of endogenous purine metabolism, maybe also play a important role. Luca's study showed both inflammation and oxidative stress have been shown to cause vascular endothelial damage, which is implicated in the occurrence and progression of various diseases, including vascular depression and dementia.[20]serum uric acid can cause inflammation and oxidative stress, so we can infer that the level of serum uric acid is connect with vascular inhibition.

This study has several advantages. First of all, there is a paucity of research examining the gender-specific connection between serum uric acid and depression.

Secondly, a scarcity of studies has delved into the communication of serum uric acid with various factors, including age, education level, activity, smoking, drinking, income, creatinine, race/ethnicity, body mass index, and albumin-to-creatinine ratio, in relation to depression prevalence. Thirdly, the study utilizes the NHANES dataset, which employs a complex, multi-stage probabilistic sampling design, thus enhancing the significance of our findings. However, there are also some limitations to consider. Firstly, the cross-sectional nature of the study precludes any definitive conclusions regarding causality between serum uric acid and depression. Secondly, the individuals that investigated were exclusively midlife and senior Americans, limiting the generalizability of these findings to other populations. Finally, depression was assessed using the PHQ-9 questionnaire, which may introduce minor inaccuracies.

Conclusion

In summary, our research shows that high serum uric acid level might reduce the incidence of depression in men, but serum uric acid is independent risk factors of depression among female patients, and people with diabetes also increase the risk of depression for both men and women. This finding will provide a good reference for the uric acid regulation of patients with depression and diabetes. At all events, the gender connection between serum uric acid level and depression still needs to be further explored.

Funding

None

Conflict of Interest

None

Data Availability

The NHANES database is publicly available (http://www.cdc.gov/nchs/nhanes.htm).

Reference

1. Ames BN, Cathcart R, Schwiers E, Hochstein P. Uric acid provides an antioxidant defense in humans against oxidant- and radical-caused aging and cancer: a hypothesis. Proc Natl Acad Sci U S A. 1981;78(11):6858-62.

2. Kirschbaum B. Correlation studies of plasma paraoxonase activity and uric acid concentration with AAPH-Induced erythrocyte hemolysis in hemodialysis patients. Artif Organs. 2004;28(3):259-64.

3. Halliwell B. Oxidative stress and neurodegeneration: where are we now? J Neurochem. 2006;97(6):1634-58.

4. Kivity S, Kopel E, Maor E, Abu-Bachar F, Segev S, Sidi Y, et al. Association of serum uric acid and cardiovascular disease in healthy adults. Am J Cardiol.2013;111(8):1146-51.

5. Nagahama K, Inoue T, Kohagura K, Kinjo K, Ohya Y. Associations between serum uric acid levels and the incidence of hypertension and metabolic syndrome: a 4-year follow-up study of a large screened cohort in Okinawa, Japan. Hypertens Res. 2015;38(3):213-8.

6. Dai X, Yuan J, Yao P, Yang B, Gui L, Zhang X, et al. Association between serum uric acid and the metabolic syndrome among a middle- and old-age Chinese population. Eur J Epidemiol. 2013;28(8):669-76.

7. Lyngdoh T, Bochud M, Glaus J, Castelao E, Waeber G, Vollenweider P, et al. Associations of serum uric acid and SLC2A9 variant with depressive and anxiety

disorders: a population-based study. PloS One. 2013;8(10):e76336.

8. Vannorsdall TD, Kueider AM, Carlson MC, Schretlen DJ. Higher baseline serum uric acid is associated with poorer cognition but not rates of cognitive decline in women. Exp Gerontol. 2014;60:136-9.

9. Lei X, Sun X, Strauss J, Zhang P, Zhao Y. Depressive symptoms and SES among the mid-aged and elderly in China: evidence from the China Health and Retirement Longitudinal Study national baseline. Soc Sci Med. 2014;120:224-32.

10. Li Y, Zhao L, Yu D, Ding G. Associations between serum uric acid and depression among middle-aged and elderly participants in China. Psychol Health Med. 2019;24(10):1277-86.

11. Tao R, Li H. High serum uric acid level in adolescent depressive patients. J Affect Disord. 2015;174:464-6.

12. Meng X, Huang X, Deng W, Li J, Li T. Serum uric acid a depression biomarker. PloS One. 2020;15(3):e0229626.

13. Zitt E, Fischer A, Lhotta K, Concin H, Nagel G. Sex- and age-specific variations, temporal trends and metabolic determinants of serum uric acid concentrations in a large population-based Austrian cohort. Sci Rep.2020;10(1):7578.

14. Kim JO, Park GN, Oh JW, Lee S. Association between uric acid and depressive symptoms in older adults: The Korea National Health and Nutrition Examination Survey. Int J Geriatr Psychiatry. 2023;38(7):e5963.

15. Jakše B, Jakše B, Pajek M, Pajek J. Uric Acid and Plant-Based Nutrition. Nutrients. 2019;11(8).

16. Joosten LAB, Crişan TO, Bjornstad P, Johnson RJ. Asymptomatic hyperuricaemia: a silent activator of the innate immune system. Nat Rev Rheumatol.2020;16(2):75-86.

17. Bowman GL, Shannon J, Frei B, Kaye JA, Quinn JF. Uric acid as a CNS antioxidant. J Alzheimers Dis: JAD. 2010;19(4):1331-6.

18. Ortiz R, Ulrich H, Zarate CA, Jr., Machado-Vieira R. Purinergic system dysfunction in mood disorders: a key target for developing improved therapeutics. Prog Neuropsychopharmacol Biol Psychiatry. 2015;57:117-31.

19. Domenici MR, Ferrante A, Martire A, Chiodi V, Pepponi R, Tebano MT, et al. Adenosine A(2A) receptor as potential therapeutic target in neuropsychiatric disorders. Pharmacol Res. 2019;147:104338.

20. Luca M, Luca A. Oxidative Stress-Related Endothelial Damage in Vascular Depression and Vascular Cognitive Impairment: Beneficial Effects of Aerobic Physical Exercise. Oxid Med Cell Longev. 2019;2019:8067045.

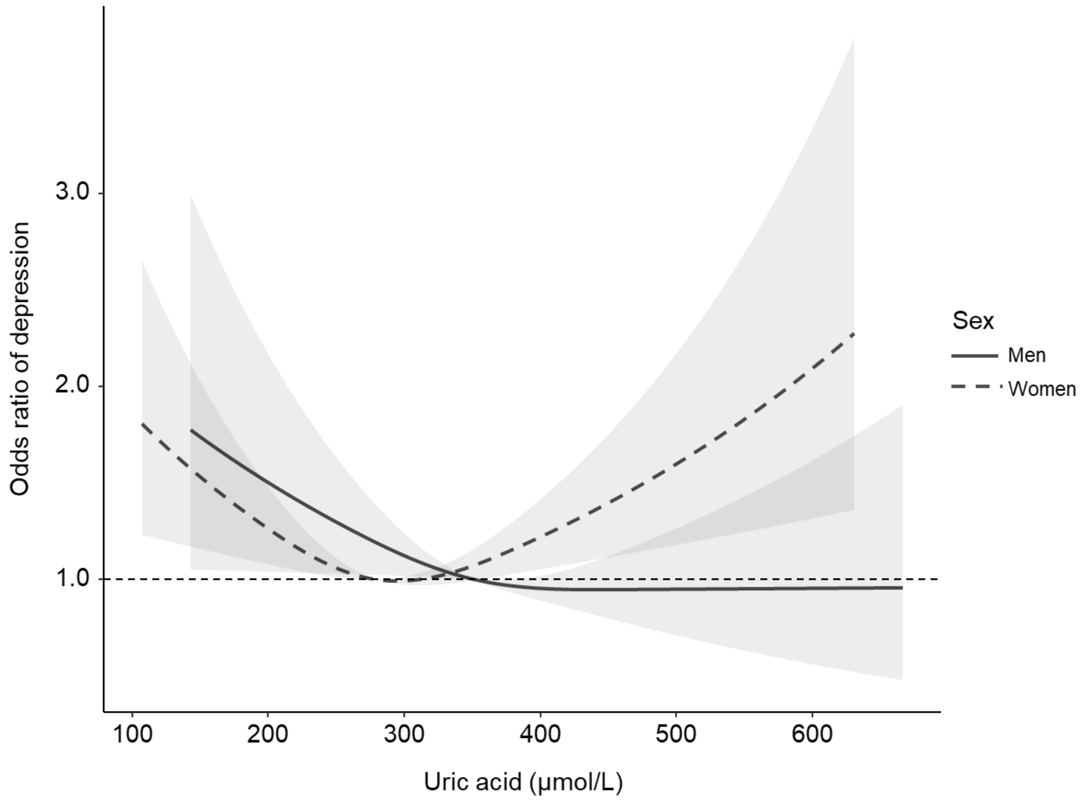

Figure 1. Adjusted cubic splines for the odds ratio of depression by uric acid levels in men and women

Model was adjusted for age, education level, physical activity level, smoking status, drinking status, income, creatinine, race/ethnicity, body mass index, and albumin-to-creatinine ratio

Table 1. The baseline characteristics of included subjects

Variables	Endorsing depression symptoms（male/female）	Not endorsing depression symptoms（male/female）
N %（95%CI）	6.25(5.56-6.94) / 4.32(3.82-4.81)	93.74(93.06-94.44) / 95.68(95.19-96.18)
Uric acid（umol/L）	358.05(350.58-365.51) / 292.94(284.90-300.98)	361.60(359.27-363.94) / 283.04(281.25-284.83)
race %（95%CI）		
race1	9.71(6.93-12.49) / 7.91(5.07-10.76)	10.43(8.44-12.43) / 8.59(6.89-10.29)
race2	8.10(5.79-10.40) / 10.20(7.23-13.17)	5.92(4.78-7.07) / 5.82(4.65-6.32)
race3	62.89(57.33-68.43) / 16.47(12.66-20.28)	65.26(61.85-68.67) / 60.48(54.10-66.86)
race4	12,70(9.50-15.92) / 16.47(12.66-20.28)	10.61(8.44-12.43) / 12.24(10.31-14.17)
race5	6.60(4.37-8.82 / 4.93(2.43-7.43)	7.78(6.67-8.89) / 7.42(6.50-8.33)
Age，year（95%CI）	42.20（40.95-43.45）/ 44.53（42.79-46.26）	42.55（42.00-43.10）/ 45.41（44.79-46.03）
education，%（95%CI）		
education1	8.13(5.78-10.48) / 8.96(6.07-11.85)	5.21(4.45-5.96) / 4.28(4.18-5.38)
education2	15.88(12.87-18.90) / 21.82(17.06-26.58)	11.47(10.22-12.72) / 9.93(8.85-11.00)
education3	28.30(23.55-33.05) / 23,64(18.22-29.06)	23.05(21.65-24.44) / 20.89(19.56-22.21)
education4	32.95(26.90-39.00) / 33.02(26.83-39.21)	29.75(28.56-30.93) / 33.73(32.27-35.20)
education5	14.74(9.52-19.95) / 12.57(8.51-16.63)	30.46(28.20-32.71) / 30.63(28.24-33.02)
income （95%CI）	9.87（8.67-10.98）/ 8.96(7.68-10.25)	11.94（11.53-12.35）/ 11.48(11.06-11.90)
Smoking,% （95%CI）		
yes	63.88(58.93-68.82) / 59.79（54.85-64.74）	47.38(45.73-49.02) / 35.62(33.99-37.25)
no	36.12(31.18-41.06) / 40.21(35.26-45.15)	52.58(50.94-54.23) / 64.31(62.70-65.92)
Drinking,%（95%CI）		
yes	83.27(79.54-87.00) / 70.36(65.29-75.42)	84.68(83.89-86.42) / 68.21(65.91-70.52)
no	16.60(12.90-20.30) / 29.08(24.23-33.93)	15.24(13.51-16.98) / 31.74(29.43-34.05)
met-activity(95%CI)	5650.216(3101.264-8199.169) / 2257.20(1763.15-2751.26)	5841.14(5406.086-6276.194) / 2959.90(2670.36-3249.44)
BMI (kg/m2)		
Systolic blood pressure (mmHg) (95%CI)	123.96（121.93-125.99）/ 119.79(117.75-121.84)	122.65（122.16-123.13）/ 119.11(118.59-119.63)
Diastolic blood pressure (mmHg) (95%CI)	73.08（71.94-74.21）/ 70.17(68.65-71.68)	71.63（71.04-72.22）/ 68.78(68.26-69.30)
HbA1c(%) (95%CI)	5.63(5.55-5.71) / 5.71(5.61-5.81)	5.58(5.55-5.61) / 5.56(5.53-5.58)
Plasma glucose:(mmol/L) (95%CI)	6.04(5.83-6.26) / 6.06	5.98(5.92-6.04) / 5.80-6.32)
Insulin (uU/mL) (95%CI)	15.06(13.24-16.88) / 15.45(13.71-17.20)	13.66(12.99-14.33) / 12.11(11.71-12.51)
ogtt(mmol/L) (95%CI)	6.37(5.93-6.81) / 6.84(6.34-7.34)	6.18(6.07-6.29) / 6.43(6.32-6.53)
TG (mg/dL) (95%CI)	1.85(1.63-2.07) / 1.49(1.34-1.65)	1.52(1.47-1.58) / 1.26(1.22-1.30)
LDL (mg/dL) (95%CI)	3.01(2.90-3.13) / 3.06(2.86-3.25)	2.98(2.94-3.01) / 2.95(2.91-2.98)
AST (u/L) (95%CI)	28.39(26.68-30.09) / 26,59(22.87-30.30)	27.70(27.35-28.09) / 27.70(27.35-28.09)
ALT (u/L) (95%CI)	31.27(29.26-33.27) / 23.95(21.91-25.99)	30.22(29.67-30.77) / 21.04(20.71-21.37)
GGT(u/L) (95%CI)	38.70(34.16-43.24) / 32.65(27.32-37.97)	30.56(29.76-31.36) / 22.29(21.24-23.33)
cr	0.98(0.95-1.01) / 0.80(0.76-0.84)	0.97(0.97-0.98) / 0.76(0.76-0.77)
acr	45.81(26.86-64.76) / 52.06(18.16-85.97)	26.05(20.75-31.34) / 27.22(23.04-31.40)
prediagDM2, % (95%CI)		
yes	7.98(6.11-9.86) / 13.97(10.59-17.34)	7.21(6.53-7.88) / 7.16(6.46-7.87)
no	88.00(85.39-90.61) / 82.18(77.97-86.39)	91.12(90.40-91.83) / 90.99(90.22-91.75)

Table 2. The association between uric acid categories and depression by gender and diabetes status

Uric acid category	Diabetes			Non-diabetes			Total		
	Case/total	Unadjusted OR (95%CI)	Adjusted OR (95%CI)	Case/total	Unadjusted OR (95%CI)	Adjusted OR (95%CI)	Case/total	Unadjusted OR (95%CI)	Adjusted OR (95%CI)
Men									
Tertile 1 (< 327.1 µmol/L)	40 / 553	1.00 (Ref)	1.00 (Ref)	188 / 2904	1.00 (Ref)	1.00 (Ref)	228 / 3464	1.00 (Ref)	1.00 (Ref)
Tertile 2 (327.1 ~ 386.6 µmol/L)	26 / 400	0.62 (0.29-1.32)	0.50 (0.19-1.31)	177 / 3228	0.88 (0.66-1.18)	0.91 (0.62-1.33)	203 / 3631	0.84 (0.63-1.11)	0.84 (0.58-1.22)
Tertile 3 (≥ 386.6 µmol/L)	33 / 379	0.89 (0.49-1.64)	0.72 (0.36-1.42)	158 / 2923	0.95 (0.73-1.25)	0.95 (0.64-1.41)	191 / 3305	0.93 (0.73-1.19)	0.87 (0.62-1.22)
Women									
Tertile 1 (< 249.8 µmol/L)	45 / 331	1.72 (0.92-3.22)	1.12 (0.30-4.90)	312 / 3202	0.91 (0.54-1.52)	1.01 (0.40-2.59)	358 / 3538	0.95 (0.54-1.67)	1.04 (0.39-2.78)
Tertile 2 (249.8 ~ 303.3 µmol/L)	48 / 262	1.00 (Ref)	1.00 (Ref)	289 / 3037	1.00 (Ref)	1.00 (Ref)	337 / 3306	1.00 (Ref)	1.00 (Ref)
Tertile 3 (≥ 303.3 µmol/L)	126 / 717	**1.97 (1.24-3.13)**	**1.79 (1.65-1.95)**	289 / 2835	1.10 (0.81-1.50)	0.95 (0.71-1.27)	418 / 3560	1.27 (1.00-1.61)	1.05 (0.92-1.19)

Model was adjusted for age, education level, physical activity level, smoking status, drinking status, income, creatinine, race/ethnicity, body mass index, and albumin-to-creatinine ratio

Table 3. The odds ratios of the associations between uric acid categories and each item in PHQ-9 diabetes status in men with tertile 1 of uric acid (<327.1 µmol/L) as reference

	Diabetes		Non-diabetes		Total	
	Tertile 2 (327.1 ~ 386.6 µmol/L)	Tertile 3 (≥386.6 µmol/L)	Tertile 2 (327.1 ~ 386.6 µmol/L)	Tertile 3 (≥386.6 µmol/L)	Tertile 2 (327.1 ~ 386.6 µmol/L)	Tertile 3 (≥386.6 µmol/L)
Q1: Have little interest in doing things	**0.49 (0.25-0.94)**	0.76 (0.31-1.88)	0.74 (0.54-1.02)	0.77 (0.58-1.03)	**0.71 (0.52-0.97)**	0.75 (0.57-1.00)
Q2: Feeling down, depressed, or hopeless	0.65 (0.28-1.53)	0.69 (0.32-1.49)	0.72 (0.51-1.06)	0.83 (0.57-1.21)	0.73 (0.51-1.05)	0.80 (0.56-1.14)
Q3: Trouble sleeping or sleeping too much	**0.44 (0.26-0.77)**	0.98 (0.53-1.81)	0.76 (0.56-1.04)	0.95 (0.70-1.28)	**0.72 (0.53-0.96)**	0.93 (0.72-1.21)
Q4: Feeling tired or having little energy	0.91 (0.48-1.70)	1.20 (0.69-2.07)	0.88 (0.67-1.16)	0.86 (0.64-1.15)	0.88 (0.68-1.14)	0.86 (0.66-1.12)
Q5: Poor appetite or overeating	0.38 (0.16-0.90)	0.56 (0.29-1.08)	0.92 (0.67-1.26)	1.09 (0.79-1.49)	0.81 (0.62-1.07)	0.97 (0.75-1.26)
Q6: Feeling bad about yourself	**0.34 (0.12-0.95)**	0.38 (0.14-1.01)	0.95 (0.67-1.34)	0.85 (0.58-1.25)	0.88 (0.64-1.21)	0.78 (0.56-1.09)
Q7: Trouble concentrating on things	**0.26 (0.09-0.71)**	0.52 (0.18-1.45)	0.85 (0.57-1.27)	0.89 (0.61-1.29)	0.75 (0.51-1.11)	0.79 (0.53-1.18)
Q8: Moving or speaking slowly or too fast	0.90 (0.33-2.45)	0.62 (0.28-1.34)	0.72 (0.45-1.15)	0.72 (0.43-1.22)	0.75 (0.51-1.09)	0.69 (0.45-1.05)
Q9: Thought you would be better off dead	0.33 (0.00-46.40)	0.06 (0.00-2.65)	0.92 (0.67-1.26)	0.76 (0.20-2.81)	0.83 (0.39-1.77)	0.63 (0.22-1.81)

Model was adjusted for age, education level, physical activity level, smoking status, drinking status, income, creatinine, race/ethnicity, body mass index, and albumin-to-creatinine ratio

Table 4. The odds ratios of the associations between uric acid categories and each item in PHQ-9 diabetes status in women with tertile 2 of uric acid (249.8 ~ 303.3 µmol/L) as reference

	Diabetes		Non-diabetes		Total	
	Tertile 1 (<249.8 µmol/L)	Tertile 3 (>=303.3 µmol/L)	Tertile 1 (<249.8 µmol/L)	Tertile 3 (>=303.3 µmol/L)	Tertile 1 (<249.8 µmol/L)	Tertile 3 (>=303.3 µmol/L)
Q1: Have little interest in doing things	1.22 (0.19-7.75)	1.30 (0.84-2.01)	1.18 (0.80-1.75)	1.04 (0.36-2.99)	1.21 (0.77-1.92)	1.08 (0.55-2.13)
Q2: Feeling down, depressed, or hopeless	1.52 (0.11-20.28)	1.29 (0.50-3.34)	1.09 (0.64-1.86)	0.94 (0.41-2.15)	1.13 (0.53-2.39)	0.99 (0.47-2.06)
Q3: Trouble sleeping or sleeping too much	0.76 (0.41-1.39)	1.30 (0.66-2.55)	1.12 (0.36-3.44)	0.97 (0.50-1.88)	1.11 (0.39-3.19)	1.02 (0.66-1.58)
Q4: Feeling tired or having little energy	0.82 (0.34-1.99)	1.29 (1.06-1.55)	0.90 (0.36-2.24)	0.99 (0.95-1.03)	0.91 (0.37-2.22)	**1.05 (1.00-1.09)**
Q5: Poor appetite or overeating	0.92 (0.41-2.03)	1.32 (0.69-2.52)	0.99 (0.27-3.63)	0.87 (0.46-1.64)	0.99 (0.28-3.47)	0.92 (0.58-1.46)
Q6: Feeling bad about yourself	1.24 (0.95-1.61)	1.12 (0.31-4.03)	0.93 (0.53-1.61)	**1.04 (1.02-1.06)**	0.96 (0.58-1.58)	1.05 (0.89-1.25)
Q7: Trouble concentrating on things	1.85 (0.55-6.28)	2.28 (1.13-4.60)	0.97 (0.85-1.11)	1.01 (0.46-2.23)	1.03 (0.85-1.26)	1.11 (0.57-2.15)
Q8: Moving or speaking slowly or too fast	1.13 (0.32-4.08)	1.02 (0.95-1.10)	1.05 (0.88-1.27)	1.24 (0.35-4.37)	1.04 (0.76-1.42)	1.19 (0.42-3.35)
Q9: Thought you would be better off dead	**0.40 (0.21-0.74)**	0.64 (0.12-3.34)	1.02 (0.26-3.96)	1.55 (0.95-2.52)	0.89 (0.17-4.58)	1.35 (0.56-3.25)

Model was adjusted for age, education level, physical activity level, smoking status, drinking status, income, creatinine, race/ethnicity, body mass index, and albumin-to-creatinine ratio

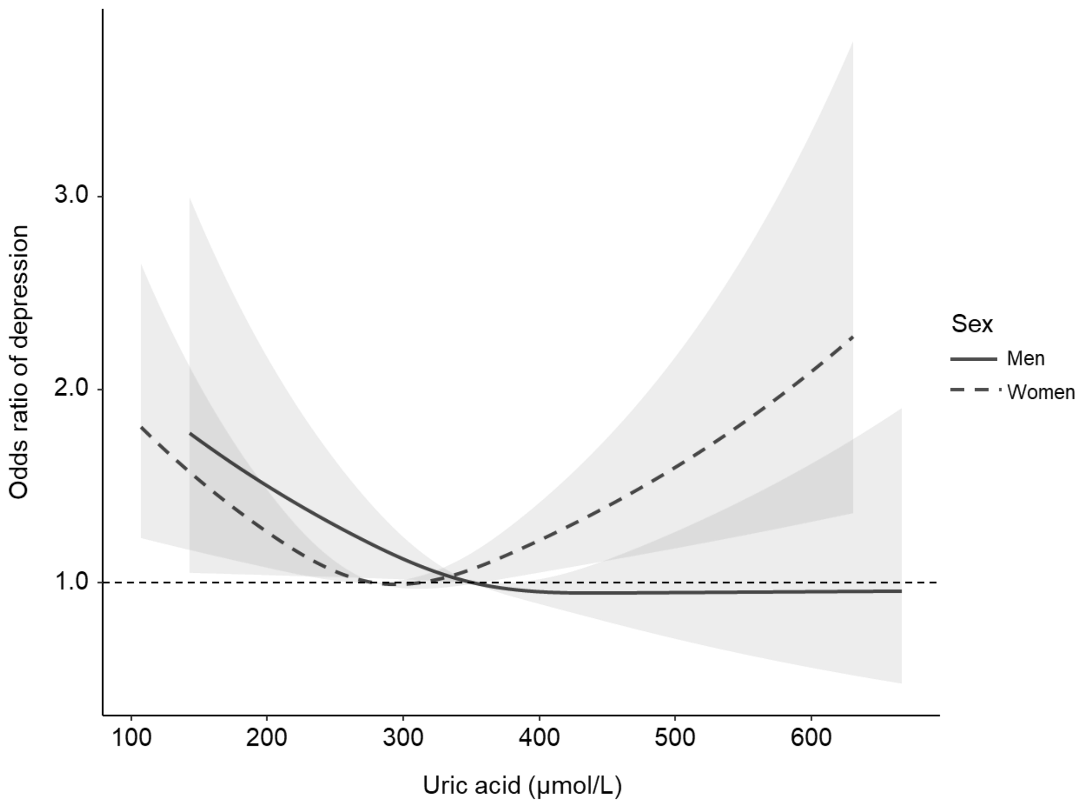

Figure 1. Adjusted cubic splines for the odds ratio of depression by uric acid levels in men and women

Model was adjusted for age, education level, physical activity level, smoking status, drinking status, income, creatinine, race/ethnicity, body mass index, and albumin-to-creatinine ratio

Table 1. The baseline characteristics of included subjects

Variables	Endorsing depression symptoms（male/female）	Not endorsing depression symptoms（male/female）
N %（95%CI）	6.25(5.56-6.94) / 4.32(3.82-4.81)	93.74(93.06-94.44) / 95.68(95.19-96.18)
Uric acid（umol/L）	358.05(350.58-365.51) / 292.94(284.90-300.98)	361.60(359.27-363.94) / 283.04(281.25-284.83)
race %（95%CI）		
race1	9.71(6.93-12.49) / 7.91(5.07-10.76)	10.43(8.44-12.43) / 8.59(6.89-10.29)
race2	8.10(5.79-10.40) / 10.20(7.23-13.17)	5.92(4.78-7.07) / 5.82(4.65-6.32)
race3	62.89(57.33-68.43) / 16.47(12.66-20.28)	65.26(61.85-68.67) / 60.48(54.10-66.86)
race4	12,70(9.50-15.92) / 16.47(12.66-20.28)	10.61(8.44-12.43) / 12.24(10.31-14.17)
race5	6.60(4.37-8.82 / 4.93(2.43-7.43)	7.78(6.67-8.89) / 7.42(6.50-8.33)
Age，year（95%CI）	42.20（40.95-43.45）/ 44.53（42.79-46.26）	42.55（42.00-43.10）/ 45.41（44.79-46.03）
education，%（95%CI）		
education1	8.13(5.78-10.48) / 8.96(6.07-11.85)	5.21(4.45-5.96) / 4.28(4.18-5.38)
education2	15.88(12.87-18.90) / 21.82(17.06-26.58)	11.47(10.22-12.72) / 9.93(8.85-11.00)
education3	28.30(23.55-33.05) / 23,64(18.22-29.06)	23.05(21.65-24.44) / 20.89(19.56-22.21)
education4	32.95(26.90-39.00) / 33.02(26.83-39.21)	29.75(28.56-30.93) / 33.73(32.27-35.20)
education5	14.74(9.52-19.95) / 12.57(8.51-16.63)	30.46(28.20-32.71) / 30.63(28.24-33.02)
income （95%CI）	9.87（8.67-10.98）/ 8.96(7.68-10.25)	11.94（11.53-12.35）/ 11.48(11.06-11.90)
Smoking,% （95%CI）		
yes	63.88(58.93-68.82) / 59.79（54.85-64.74）	47.38(45.73-49.02) / 35.62(33.99-37.25)
no	36.12(31.18-41.06) / 40.21(35.26-45.15)	52.58(50.94-54.23) / 64.31(62.70-65.92)
Drinking,%（95%CI）		
yes	83.27(79.54-87.00) / 70.36(65.29-75.42)	84.68(83.89-86.42) / 68.21(65.91-70.52)
no	16.60(12.90-20.30) / 29.08(24.23-33.93)	15.24(13.51-16.98) / 31.74(29.43-34.05)
met-activity(95%CI)	5650.216(3101.264-8199.169) / 2257.20(1763.15-2751.26)	5841.14(5406.086-6276.194) / 2959.90(2670.36-3249.44)
BMI (kg/m2)		
Systolic blood pressure (mmHg) (95%CI)	123.96（121.93-125.99）/ 119.79(117.75-121.84)	122.65（122.16-123.13）/ 119.11(118.59-119.63)
Diastolic blood pressure (mmHg) (95%CI)	73.08（71.94-74.21）/ 70.17(68.65-71.68)	71.63（71.04-72.22）/ 68.78(68.26-69.30)
HbA1c(%) （95%CI）	5.63(5.55-5.71) / 5.71(5.61-5.81)	5.58(5.55-5.61) / 5.56(5.53-5.58)
Plasma glucose:(mmol/L) (95%CI)	6.04(5.83-6.26) / 6.06	5.98(5.92-6.04) / 5.80-6.32)
Insulin (uU/mL) (95%CI)	15.06(13.24-16.88) / 15.45(13.71-17.20)	13.66(12.99-14.33) / 12.11(11.71-12.51)
ogtt(mmol/L) (95%CI)	6.37(5.93-6.81) / 6.84(6.34-7.34)	6.18(6.07-6.29) / 6.43(6.32-6.53)
TG (mg/dL) (95%CI)	1.85(1.63-2.07) / 1.49(1.34-1.65)	1.52(1.47-1.58) / 1.26(1.22-1.30)
LDL (mg/dL) (95%CI)	3.01(2.90-3.13) / 3.06(2.86-3.25)	2.98(2.94-3.01) / 2.95(2.91-2.98)
AST (u/L) (95%CI)	28.39(26.68-30.09) / 26,59(22.87-30.30)	27.70(27.35-28.09) / 27.70(27.35-28.09)
ALT (u/L) (95%CI)	31.27(29.26-33.27) / 23.95(21.91-25.99)	30.22(29.67-30.77) / 21.04(20.71-21.37)
GGT(u/L) (95%CI)	38.70(34.16-43.24) / 32.65(27.32-37.97)	30.56(29.76-31.36) / 22.29(21.24-23.33)
cr	0.98(0.95-1.01) / 0.80(0.76-0.84)	0.97(0.97-0.98) / 0.76(0.76-0.77)
acr	45.81(26.86-64.76) / 52.06(18.16-85.97)	26.05(20.75-31.34) / 27.22(23.04-31.40)
prediagDM2, % (95%CI)		
yes	7.98(6.11-9.86) / 13.97(10.59-17.34)	7.21(6.53-7.88) / 7.16(6.46-7.87)
no	88.00(85.39-90.61) / 82.18(77.97-86.39)	91.12(90.40-91.83) / 90.99(90.22-91.75)

Table 2. The association between uric acid categories and depression by gender and diabetes status

	Diabetes			Non-diabetes			Total		
Uric acid category	Case/total	Unadjusted OR (95%CI)	Adjusted OR (95%CI)	Case/total	Unadjusted OR (95%CI)	Adjusted OR (95%CI)	Case/total	Unadjusted OR (95%CI)	Adjusted OR (95%CI)
Men									
Tertile 1 (< 327.1 μmol/L)	40 / 553	1.00 (Ref)	1.00 (Ref)	188 / 2904	1.00 (Ref)	1.00 (Ref)	228 / 3464	1.00 (Ref)	1.00 (Ref)
Tertile 2 (327.1 ~ 386.6 μmol/L)	26 / 400	0.62 (0.29-1.32)	0.50 (0.19-1.31)	177 / 3228	0.88 (0.66-1.18)	0.91 (0.62-1.33)	203 / 3631	0.84 (0.63-1.11)	0.84 (0.58-1.22)
Tertile 3 (≥ 386.6 μmol/L)	33 / 379	0.89 (0.49-1.64)	0.72 (0.36-1.42)	158 / 2923	0.95 (0.73-1.25)	0.95 (0.64-1.41)	191 / 3305	0.93 (0.73-1.19)	0.87 (0.62-1.22)
Women									
Tertile 1 (< 249.8 μmol/L)	45 / 331	1.72 (0.92-3.22)	1.12 (0.30-4.90)	312 / 3202	0.91 (0.54-1.52)	1.01 (0.40-2.59)	358 / 3538	0.95 (0.54-1.67)	1.04 (0.39-2.78)
Tertile 2 (249.8 ~ 303.3 μmol/L)	48 / 262	1.00 (Ref)	1.00 (Ref)	289 / 3037	1.00 (Ref)	1.00 (Ref)	337 / 3306	1.00 (Ref)	1.00 (Ref)
Tertile 3 (≥ 303.3 μmol/L)	126 / 717	**1.97 (1.24-3.13)**	**1.79 (1.65-1.95)**	289 / 2835	1.10 (0.81-1.50)	0.95 (0.71-1.27)	418 / 3560	1.27 (1.00-1.61)	1.05 (0.92-1.19)

Model was adjusted for age, education level, physical activity level, smoking status, drinking status, income, creatinine, race/ethnicity, body mass index, and albumin-to-creatinine ratio

Table 3. The odds ratios of the associations between uric acid categories and each item in PHQ-9 diabetes status in men with tertile 1 of uric acid (<327.1 μmol/L) as reference

	Diabetes		Non-diabetes		Total	
	Tertile 2 (327.1 ~ 386.6 μmol/L)	Tertile 3 (≥386.6 μmol/L)	Tertile 2 (327.1 ~ 386.6 μmol/L)	Tertile 3 (≥386.6 μmol/L)	Tertile 2 (327.1 ~ 386.6 μmol/L)	Tertile 3 (≥386.6 μmol/L)
Q1: Have little interest in doing things	**0.49 (0.25-0.94)**	0.76 (0.31-1.88)	0.74 (0.54-1.02)	0.77 (0.58-1.03)	**0.71 (0.52-0.97)**	0.75 (0.57-1.00)
Q2: Feeling down, depressed, or hopeless	0.65 (0.28-1.53)	0.69 (0.32-1.49)	0.72 (0.51-1.06)	0.83 (0.57-1.21)	0.73 (0.51-1.05)	0.80 (0.56-1.14)
Q3: Trouble sleeping or sleeping too much	**0.44 (0.26-0.77)**	0.98 (0.53-1.81)	0.76 (0.56-1.04)	0.95 (0.70-1.28)	**0.72 (0.53-0.96)**	0.93 (0.72-1.21)
Q4: Feeling tired or having little energy	0.91 (0.48-1.70)	1.20 (0.69-2.07)	0.88 (0.67-1.16)	0.86 (0.64-1.15)	0.88 (0.68-1.14)	0.86 (0.66-1.12)
Q5: Poor appetite or overeating	0.38 (0.16-0.90)	0.56 (0.29-1.08)	0.92 (0.67-1.26)	1.09 (0.79-1.49)	0.81 (0.62-1.07)	0.97 (0.75-1.26)
Q6: Feeling bad about yourself	**0.34 (0.12-0.95)**	0.38 (0.14-1.01)	0.95 (0.67-1.34)	0.85 (0.58-1.25)	0.88 (0.64-1.21)	0.78 (0.56-1.09)
Q7: Trouble concentrating on things	**0.26 (0.09-0.71)**	0.52 (0.18-1.45)	0.85 (0.57-1.27)	0.89 (0.61-1.29)	0.75 (0.51-1.11)	0.79 (0.53-1.18)
Q8: Moving or speaking slowly or too fast	0.90 (0.33-2.45)	0.62 (0.28-1.34)	0.72 (0.45-1.15)	0.72 (0.43-1.22)	0.75 (0.51-1.09)	0.69 (0.45-1.05)
Q9: Thought you would be better off dead	0.33 (0.00-46.40)	0.06 (0.00-2.65)	0.92 (0.67-1.26)	0.76 (0.20-2.81)	0.83 (0.39-1.77)	0.63 (0.22-1.81)

Model was adjusted for age, education level, physical activity level, smoking status, drinking status, income, creatinine, race/ethnicity, body mass index, and albumin-to-creatinine ratio

Table 4. The odds ratios of the associations between uric acid categories and each item in PHQ-9 diabetes status in women with tertile 2 of uric acid (249.8 ~ 303.3 μmol/L) as reference

	Diabetes		Non-diabetes		Total	
	Tertile 1 (<249.8 μmol/L)	Tertile 3 (>=303.3 μmol/L)	Tertile 1 (<249.8 μmol/L)	Tertile 3 (>=303.3 μmol/L)	Tertile 1 (<249.8 μmol/L)	Tertile 3 (>=303.3 μmol/L)
Q1: Have little interest in doing things	1.22 (0.19-7.75)	1.30 (0.84-2.01)	1.18 (0.80-1.75)	1.04 (0.36-2.99)	1.21 (0.77-1.92)	1.08 (0.55-2.13)
Q2: Feeling down, depressed, or hopeless	1.52 (0.11-20.28)	1.29 (0.50-3.34)	1.09 (0.64-1.86)	0.94 (0.41-2.15)	1.13 (0.53-2.39)	0.99 (0.47-2.06)
Q3: Trouble sleeping or sleeping too much	0.76 (0.41-1.39)	1.30 (0.66-2.55)	1.12 (0.36-3.44)	0.97 (0.50-1.88)	1.11 (0.39-3.19)	1.02 (0.66-1.58)
Q4: Feeling tired or having little energy	0.82 (0.34-1.99)	1.29 (1.06-1.55)	0.90 (0.36-2.24)	0.99 (0.95-1.03)	0.91 (0.37-2.22)	**1.05 (1.00-1.09)**
Q5: Poor appetite or overeating	0.92 (0.41-2.03)	1.32 (0.69-2.52)	0.99 (0.27-3.63)	0.87 (0.46-1.64)	0.99 (0.28-3.47)	0.92 (0.58-1.46)
Q6: Feeling bad about yourself	1.24 (0.95-1.61)	1.12 (0.31-4.03)	0.93 (0.53-1.61)	**1.04 (1.02-1.06)**	0.96 (0.58-1.58)	1.05 (0.89-1.25)
Q7: Trouble concentrating on things	1.85 (0.55-6.28)	2.28 (1.13-4.60)	0.97 (0.85-1.11)	1.01 (0.46-2.23)	1.03 (0.85-1.26)	1.11 (0.57-2.15)
Q8: Moving or speaking slowly or too fast	1.13 (0.32-4.08)	1.02 (0.95-1.10)	1.05 (0.88-1.27)	1.24 (0.35-4.37)	1.04 (0.76-1.42)	1.19 (0.42-3.35)
Q9: Thought you would be better off dead	**0.40 (0.21-0.74)**	0.64 (0.12-3.34)	1.02 (0.26-3.96)	1.55 (0.95-2.52)	0.89 (0.17-4.58)	1.35 (0.56-3.25)

Model was adjusted for age, education level, physical activity level, smoking status, drinking status, income, creatinine, race/ethnicity, body mass index, and albumin-to-creatinine ratio
