# Supplementary material for: Associations between serum uric acid and depression in U.S. adults: An analysis of the national health and nutrition examination survey 2007–2016
Source: PLoS One. 2025 Apr 8;20(4):e0321387. doi: 10.1371/journal.pone.0321387 (PMC11978101; doi:10.1371/journal.pone.0321387)
Supplement: Attachment — Submitted filename: Response to Reviewers.docx [file pone.0321387.s003.docx]

Dear editor and reviewers,

We sincerely thank the editor and all reviewers for their valuable feedback that we have used to improve the quality of our manuscript. The reviewer comments are laid out below in italicized font and specific concerns have been numbered. Our response is given in normal font and changes/additions to the manuscript are given in the red text.

*Academic Editor Lai Kuan Lee:*

*1.Please include the following items when submitting your revised manuscript: 1) A rebuttal letter that responds to each point raised by the academic editor and reviewer(s). You should upload this letter as a separate file labeled 'Response to Reviewers'. 2) A marked-up copy of your manuscript that highlights changes made to the original version. You should upload this as a separate file labeled 'Revised Manuscript with Track Changes'. 3) An unmarked version of your revised paper without tracked changes. You should upload this as a separate file labeled 'Manuscript'*

Response: I have submitted the corresponding items as required.

*Reviewer #1:*

*1. Your citation of reference # 11 was only done on male adolescents. Please change the language you have used to seem to indicate the relationship does not exist in women when what you wanted to convey was that there was no data on women.
2. In results, The paragraph starting with "As shown in Figure 1,..." could use a change in grammar and syntax to better explain the chart. (e.g- whether serum uric acid goes up or down, it increases the risk of depression in women"). The relationship between different tertiles and the depression incidence could be explained better as well. There would be a better way of explaining the general relationship and the one when diabetes is involved separately.
3. The same in the Discussion section- "Higher serum uric acid has a correlation with a higher risk of depression in American female population, but it's opposite in male". That's partially true but can be described in a better way.
4. Why is it surprising that men show lower prevalence of depression with higher level of serum uric acid (isn't that what previous data has shown?)*

Response: We appreciate your attention to the linguistic aspects of our manuscript. We understand that clear and concise communication is essential for scientific writing. In response, we have carefully revised the language, paying attention to sentence structure, vocabulary choices, and tone. And here we did not list the changes but marked in red in the revised paper. We believe that these improvements will make our paper more accessible and engaging for readers.

*5. There is a typo of "Man" for "men" as well. Next paragraph in Discussion has typo , also "it's similar to our found" is a typo.*

Response: Thank you for pointing out the spelling errors in our manuscript. We have carefully gone through the entire corrected all the identified spelling document and mistakes. We apologize for any inconvenience caused and are committed to ensuring the highest level of quality in our future submissions.

6. *The conclusion might also need to be rewritten with correction of typos ("uric acid is independent risk factors", "people with diabetes also increase the risk of depression..."). Also, adding diabetes as a risk factor for depression seems unrelated to your study about uric acid. If it is not, please explain it.*

Response: We have rewritten the conclusion according to the Reviewer' suggestion. P11, Line189-197.

7. *Then in Discussion you mention other studies that show results opposite or not in keeping with your results which brings up a big question about the veracity and reproducibility of these results. There are many confounding factors that can lead to uric acid increase over the ones related to depression and I think the connection between the two especially in a cross sectional data does not explain much. Please discuss other confounders in discussion. What other factors can determine uric acid levels? What other biochemical reactions cause uric acid to be formed other than purines?*

Response: We sincerely appreciate the valuable comments. We have checked the literature carefully and added more references on the influencing factors of uric acid into Discussion part in the revised manuscript.

*Reviewer #2:*

1. *abstract keywords ; should we put?..... oxidative stress; diabetes*

Response: We sincerely appreciate the valuable comments. We have changed abstract keywords to “serum uric acid; depression; gender”.

2. *Introduction: 6-7th line and 16-17th: repeated "serum uric acid contributes to approximately 60% of the antioxidant capacity in body's blood"*

Response: We feel really sorry for our careless mistakes, we have changed 16-17th to “in the extracellular chambers, serum uric acid plays a crucial role with its main function being that of an antioxidant”.

*3.* *Methods and material: Study design and subjects: last line: do 20804 participants have data on uric acid and depression? as per the statement*

Response: Yes, I will upload the data on uric acid and depression.

*4.Result: 3rd line- rephrase....symptoms and illness are different. It is better to mention only :* *Depression. Was 19826 Participants score of PHQ less than 5?*

Response: We have changed “depression symptoms” to “Depression”. And 19826 Participants score of PHQ was not less than 5.

We tried our best to improve the manuscript and made some changes in the manuscript. These changes will not influence the content and framework of the paper. And here we did not list the changes but marked in red in revised paper.

We appreciate for Editors/Reviewers' warm work earnestly, and hope that the correction will meet with approval.

Once again, thank you very much for your comments and suggestions.

Yours sincerely,

JieLi

1st December, 2024.
