# Supplementary material for: Associations between serum uric acid and depression in U.S. adults: An analysis of the national health and nutrition examination survey 2007–2016
Source: PLoS One. 2025 Apr 8;20(4):e0321387. doi: 10.1371/journal.pone.0321387 (PMC11978101; doi:10.1371/journal.pone.0321387)
Supplement: Attachment — Submitted filename: Response_to_Reviewers_auresp_2.docx [file pone.0321387.s004.docx]

Dear editor and reviewers,

We sincerely thank the editor and all reviewers for their valuable feedback that we have used to improve the quality of our manuscript. The reviewer comments are laid out below in italicized font and specific concerns have been numbered. Our responses are given in normal font and changes/additions to the manuscript are given in the red text.

*1.The language and grammar mistakes in the manuscript require further polishing.*

Response: We appreciate your attention to the linguistic aspects of our manuscript. We understand that clear and concise communication is essential for scientific writing. In response, we have carefully revised the language and grammar. Our revisions are marked in red. We believe that these improvements will make our paper more accessible and engaging for readers. *2. Table 2 is being presented in a not readable format.*

Response: Thanks for your suggestion, we have re-uploaded Table 2 and adjusted its format.

*3. Please revise the authors' contributions.*

Response: We apologize for our negligence, and we have thoroughly revised the authors' contributions and completed the upload process.

4. *Ethical approval: Was the publication consent granted? Please list the human ethical approval serial number/reference code.*

Response: We sincerely appreciate your attention. With respect to the ethical review, we hereby issue the following formal statements: First, this study aims to explore the correlation between serum uric acid levels and depression, considering gender differences in U.S. adults. Second, the data come from a public database. Informed consent from patients was obtained before data inclusion. Third, this study involves no personal information. All private data are kept confidential throughout, preventing privacy breaches. It has no commercial interests. Finally, as this study complies with the Declaration of Helsinki, ethical review can be waived.

5. *Add limitation and strengths*

Response: Your suggestion is of great significance. In response, we have added the limitation and strengths

We tried our best to improve the manuscript and made some changes in the manuscript. These changes will not influence the content and framework of the paper. And here we did not list the changes but marked in red in revised paper.

We appreciate for Editors/Reviewers' warm work earnestly, and hope that the correction will meet with approval.

Once again, thank you very much for your comments and suggestions.

Yours sincerely,

JieLi

4th January, 2025.
